# Correction: Contreras-Reyes, J.E.; Cortés, D.D. Bounds on Rényi and Shannon Entropies for Finite Mixtures of Multivariate Skew-Normal Distributions: Application to Swordfish (*Xiphias gladius Linnaeus*). *Entropy* 2016, 18, 382

**DOI:** 10.3390/e22080892

**Published:** 2020-08-14

**Authors:** Javier E. Contreras-Reyes, Daniel Devia Cortés

**Affiliations:** 1 Departamento de Estadística, Facultad de Ciencias, Universidad del Bío-Bío, Concepción 4081112, Chile; 2 Instituto de Fomento Pesquero (IFOP), Blanco 839, Valparaíso 2361827, Chile; daniel.devia@ifop.cl

## Abstract

Section 3.3 of “Contreras-Reyes, J.E.; Cortés, D.D. Bounds on Rényi and Shannon Entropies for Finite Mixtures of Multivariate Skew-Normal Distributions: Application to Swordfish (*Xiphias gladius Linnaeus*). *Entropy* **2016**, *18*, 382” contains errors. Therefore, this section is retracted. However, these changes do not influence the conclusions and the other results of the paper.

The authors were not aware of one error made in the proofreading phase; therefore, we wish to make the following correction to this paper [1]:

On Equation (14), the multinomial (MN) theorem is considered [2] to obtain

(1) $$\begin{array}{ccc} \int_{R^{d}}p{\left(y;\tilde{\theta },\pi \right)}^{\alpha }dy & = & \int_{R^{d}}\sum_{k_{i}\in A}\frac{\alpha !}{k_{1}!\cdots k_{m}!}\prod_{i=1}^{m}{\left[\pi_{i}f\left(y;\theta_{i}\right)\right]}^{k_{i}}dy \end{array}$$

under the condition

(2) $$\begin{array}{ccc} \sum_{k_{i}\in A}\frac{\alpha !}{k_{1}!\cdots k_{m}!} & = & m^{\alpha }, \end{array}$$

with $A=\left\{k_{i}\in N:\;k_{i}>0,\sum_{i=1}^{m}k_{i}=\alpha ;\;i=1,\ldots ,m\right\}$, 0<α<∞, α≠1; and an *m*-component mixture model

$$p\left(y;\tilde{\theta },\pi \right)=\sum_{i=1}^{m}\pi_{i}\;f\left(y;\theta_{i}\right),$$

where $\pi_{i}\geq 0$, $\sum_{i=1}^{m}\pi_{i}=1$, $\tilde{\theta }=\left(\tilde{\xi },\tilde{\Omega },\tilde{\eta }\right)$: $\tilde{\xi }=\left(\xi_{1},\ldots ,\xi_{m}\right)$ a set of *m* location vector parameters, $\tilde{\Omega }=\left(\Omega_{1},\ldots ,\Omega_{m}\right)$ a set of *m* dispersion matrices, $\tilde{\eta }=\left(\eta_{1},\ldots ,\eta_{m}\right)$ a set of shape vector parameters, and with *m* mixing weights, $\pi =(\pi_{1},\ldots ,\pi_{m})$.

Given that integral in (1) is based on the αth-order Rényi entropy on variable y:

(3) $$R_{\alpha }\left[Y;\tilde{\theta },\pi \right]=\frac{1}{1-\alpha }\ln\int_{R^{d}}p{\left(y;\tilde{\theta },\pi \right)}^{\alpha }dy,\;0<\alpha <\infty ,\;\alpha \neq 1,$$

the condition (2) is not accomplished for any m∈N. For example, if m=2 and α=2, thus $\alpha =k_{1}+k_{2}\Rightarrow 2=1+1$ implies that $k_{i}=1$, i=1,2, because $k_{i}\in N$, $k_{i}>0$. In general, for any m∈N, the MN theorem needs $k_{i}=1$, i=1,…,m. Therefore, MN theorem can not be considered for the approximation of the Rényi entropy for any finite mixture, with αth-order satisfying 0<α<∞ and α≠1. Only for the limit α→1, the Shannon entropy is obtained as

$$H\left[Y;\tilde{\theta },\pi \right]=\lim_{\alpha \to 1}R_{\alpha }\left[Y;\tilde{\theta },\pi \right],$$

by applying l’Hôpital’s rule to $R_{\alpha }\left[Y;\tilde{\theta },\pi \right]$ with respect to α. Therefore, Lemma 3 of the paper could be considered in the limit α→1. However, this is the case of bounds on Shannon entropy addressed in Section 3.1.

In conclusion:

1. On page 1, Abstract section, the sentence “In addition, an asymptotic expression for Rényi entropy by Stirling’s approximation is given, and upper and lower bounds are reported using multinomial coefficients and some properties and inequalities of $L^{p}$ metric spaces” must to be removed.
2. On page 1, Keywords section, “frinite” should be “finite”.
3. On page 2, Paragraph 2, Introduction section, the sentences: “Additionally, an asymptotic expression for Rényi entropy due to Stirling’s approximation is given, and upper and lower bounds are reported using the multinomial coefficients and some properties and inequalities of $L^{p}$ metric spaces” and “including some theoretical results based on Stirling’s approximation and the multinomial theorem” must to be removed.
4. On pages 6–8, Section 3.3 must not be considered by the readers that need an approximation of Rényi entropy for a finite mixture of skew-normals or other densities.
5. On page 16, Paragraph 1, Section 5.1, the sentences: “We presented practical (bounds) and theoretical (bounds and asymptotic expression) results for Rényi entropy. In the case of practical results,” and “In the case of theoretical results, the bounds and approximations are based on $L^{p}$ space metric and multinomial coefficients” should be removed.
6. On page 16, Paragraph 2, Section 5.1, the sentence: “For this reason, further research should consider the exact expression and asymptotic approximation presented in this paper. For these, an algorithm to identify the multinomial coefficients restricted to conditions (15) and (18), respectively, could be developed.” should be removed.
7. On page 16, Paragraph 3, Section 5.1, the sentence: “In addition, Proposition 2 and Lemmas 1, 2 and 3” must to be modified to “In addition, Proposition 2 and Lemma 1”.
8. On pages 18–19, Appendix A, proofs of Lemmas 2 and 3 must to be removed.
9. On page 20, References section, the references [25], [38] and [39] must to be removed.
10. The reference citation number and equation number have been changed correspondingly since Section 4.

However, these changes do not influence the conclusions and the other results of the paper. The authors would like to apologize for any inconvenience caused.
